# *Sarcocystis* species: molecular identification and seroprevalence in water buffaloes (*Bubalus bubalis*)

**DOI:** 10.1186/s12917-025-04933-3

**Published:** 2025-07-22

**Authors:** Nagwa I. Toaleb, Raafat M. Shaapan, Haitham Elaadli, Kadria N. Abdel Megeed, Dina Aboelsoued

**Affiliations:** 1 Parasitology and Animal Diseases Department, Veterinary Research Institute, National Research Centre, El Buhouth St., Dokki, Giza, Egypt; 2 Zoonotic Diseases Department, Veterinary Research Institute, National Research Centre, El Buhouth St., Dokki, Giza, Egypt; 3 Department of Animal Hygiene and Zoonoses, Faculty of Veterinary Medicine, Alexandria University, Alexandria, Egypt

**Keywords:** Buffaloes, *Sarcocystis*, Molecular Analysis, SDS-PAGE, Western blot, ELISA

## Abstract

Meat infection with the coccidian protozoan zoonotic *Sarcocystis* spp. causes public health hazards and high economic loss. The current study aimed to investigate molecular identification, cyst histological examination and seroprevalence of *Sarcocystis* spp. in water buffaloes slaughtered in the main abattoirs of 6 Egyptian Governorates; Cairo, Giza, Beni-suef, Al-Sharqia, Qalyubia, and El-Beheira. Each buffalo was visually inspected for the presence of *Sarcocystis* macrocysts and blood samples were collected. Out of 900 examined water buffaloes (*Bubalus bubalis*), 246 (27.3%) were found to be infected based on macroscopic examination. Histological examination of the macrocyst revealed metrocytes close to the cyst's edge and elongated curving basophilic bradyzoites occupying most of the cyst. Depending on age, we categorized naturally infected buffaloes into 3 groups: young (< 2 y.), adult (2–5 y.) and old (5–10 y.). The highest infection rate was observed in older buffaloes aged 5–10 years, meanwhile female animals exhibited a higher prevalence of infection compared to males. The esophagus had the highest presence of *Sarcocystis* compared to other organs. Using PCR based on *18S* rRNA gene and sequencing, we isolated 3 *Sarcocystis* species from infected tissues: *S. buffalonis*, *S. fusiformis* and *S. hirsuta-*like. The prepared whole cyst antigen of *S*. *fusiformis* was characterized by 9 major polypeptides (140, 120, 78, 66, 53, 39, 32, 24 and 19 kDa) on 10% SDS-PAGE. Using western blotting, we identified 2 common immunogenic reactive bands (66 and 32 kDa) against naturally infected buffalo sera and hyperimmune rat sera. Additionally, sarcocystosis positive seroprevalence rate was 46.4% (418/900) with a sensitivity of 97.6% and specificity of 100% using indirect ELISA based on *S. fusiformis* whole cyst antigen. In conclusion, this study highlights the molecular identification, cyst histological examination and seroprevalence of *Sarcocystis* sp. among buffaloes in Egypt. ELISA using the *S. fusiformis* whole cyst antigen could be adapted to detect antibodies to *Sarcocystis* sp., in live animals, with an acceptable specificity and sensitivity.

**Supplementary Information:**

The online version contains supplementary material available at 10.1186/s12917-025-04933-3.

## Introduction

*Sarcocystis* species are intracellular zoonotic apicomplexan protozoan parasites infecting human and different domestic (buffaloes, cattle, pigs, horses, sheep, dogs, cats) and wild (birds, rodents, snakes and raccoons) animals [19, 26] with serious public health and economic significance. *Sarcocystis* tissue cyst formation takes place in the intermediate hosts and oocyst/sporocyst formation takes place in the definitive host [4]. Pathogenic species affect farm animals leading to weight loss, abortions, general weakness, reduced milk production, and mortality [26]. Also, the presence of macroscopic *Sarcocystis* cysts can lead to downgrading and condemnation of animal carcasses [22]. Sarcocystosis prevalence in adult bovine muscles could reach up to 70‒100%, in many regions of the world [9, 15].

Meat infection with different species of *Sarcocystis* can be serious for public health [9]. Water buffaloes are intermediate hosts for *Sarcocystis fusiformis*, *S. dubeyi*, *S. levinei*, *S. sinensis*, and *S. buffalonis* [48] in which muscles from the esophagus, heart, tongue and diaphragm are the target-colonizing areas of* Sarcocystis* sp. [19]. The esophagus has been found to be the most affected organ in ruminants [7, 24, 28]. In water buffaloes, sarcocystosis was detected worldwide with different infection rates ranging from 23.8 to 71.5% in Brazil [57], in The Philippines [47], in Egypt [10, 36, 55, 60] and in India [16].

Traditional methods for identification and characterization of *Sarcocystis* sp. are mostly based on isolated cyst morphology including shape and thickness of the cyst wall using gross examination and light or transmission electron microscopy [15, 20]. Antemortem diagnosis of sarcocystosis is challenging as its signs are usually similar to other diseases, and a biopsy is time-consuming and is not suitable for screening large numbers of animals before slaughtering. So, serological techniques are the method of choice for early diagnosis of sarcocystosis by the detection of specific antibodies [12]. Also, molecular methods were found to be more useful and sensitive in the detection of *Sarcocystis* spp. [37, 39].

Accurate diagnosis of *Sarcocystis* spp. is essential for evaluating their public health, veterinary, and economic impact. Therefore, this study aimed to identify *Sarcocystis* species through molecular study in combination with cyst histology and serological detection of anti-parasitic antibodies of these protozoans in tissue, cyst and serum samples.

## Materials and methods

### Study area and period

**Fig. 1:**
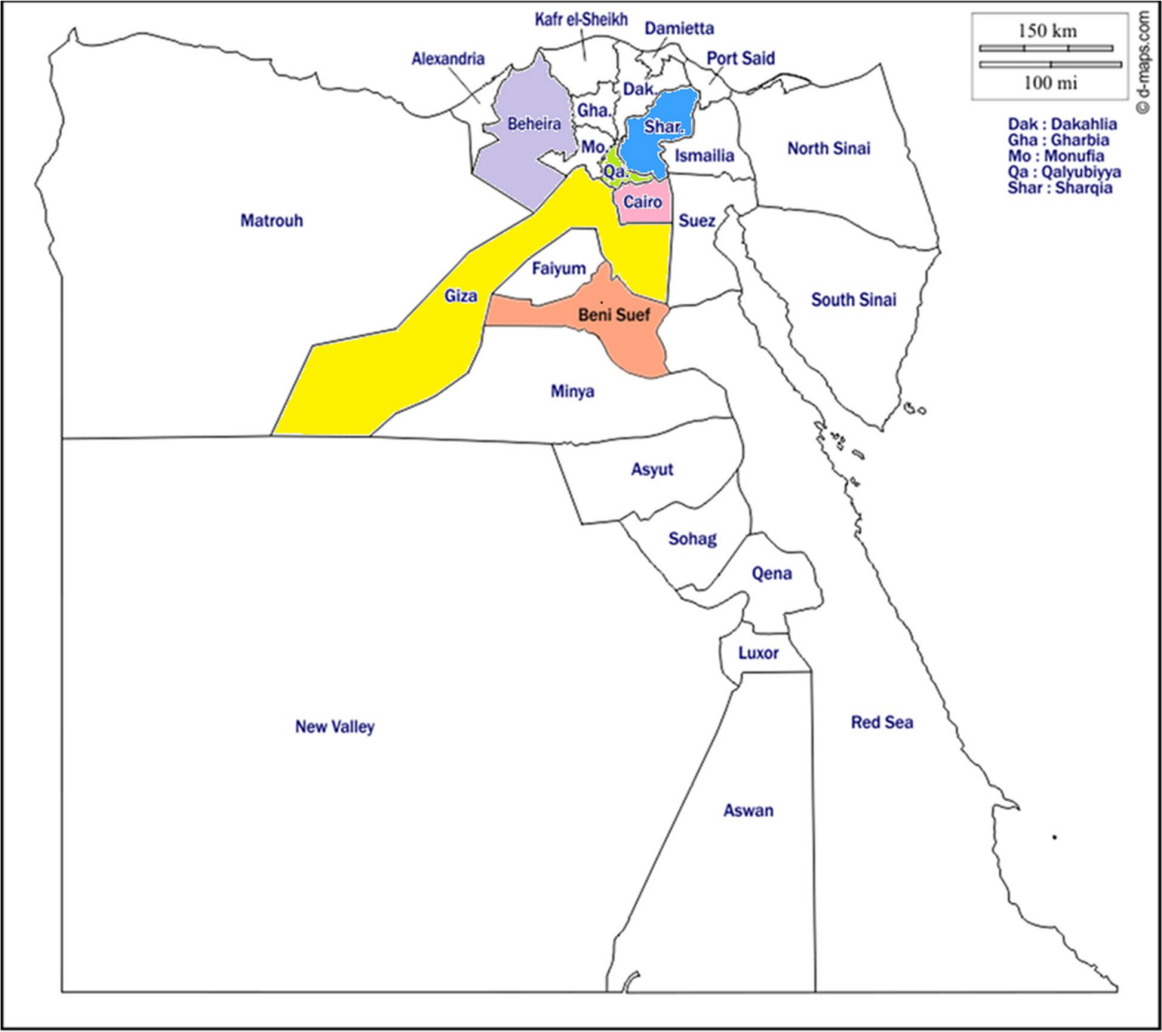
Egypt map showing Egyptian Governorates, with Cairo highlighted in pink, Giza highlighted in yellow, Beni-Suef highlighted in orange, Al-Sharqia highlighted in blue, Qalyubia highlighted in green and El-Beheira highlighted in purple to indicate where the samples were collected

Samples were collected from water buffaloes slaughtered at different localities of Egypt represented by the main economic regions: Cairo (30°2′40″N 31°14′9″E; Basatein abattoir), Giza (29.9870°N 31.2118°E; El- Moneib and El-Warak abattoirs), and the main abattoirs of Al-Sharqia (30.7°N 31.63°E), Beni-suef (29.076°N 31.097°E), Qalyubia (30.41°N 31.21°E), and El-Beheira (30.61°N 30.43°E) Governorates, Egypt (Fig. 1), during the period between January 2023 and April 2024, through several visits.

### Sample size

Sample size was calculated using the equation described by Daniel [17] with a 95% Confidence Interval (CI), 5% absolute precision [65] and 41.5% anticipated prevalence of *Sarcocystis* spp. in water buffaloes [58].

$${\varvec{n}}=\frac{{{\varvec{Z}}}^{2}\boldsymbol{ }{\varvec{p}}(1-{\varvec{p}})}{{{\varvec{d}}}^{2}}$$

where:

*n* = Required sample size, *Z* = Appropriate percentage for the standard deviation with expected CI (95%), *p* = Estimated prevalence, and *d* = anticipated absolute accuracy (5%). We examined 900 samples from slaughtered buffaloes for the occurrence of *Sarcocystis* species, with the larger sample size increasing the likelihood of finding positive cases.

### Animals

Nine hundred buffaloes from different age groups and sex were tested in this study after antemortem and postmortem inspections. We collected samples from 400 males and 500 females. Animals were grouped according to dental eruptions as young (Less than 2 years, *N* = 100, Females = 42; Males = 58), adult (2–5 years, *N* = 340, F = 175; M = 165), and old (5–10 years, *N* = 460, F = 283; M = 177).

All animals passed clinical and physical examinations before and after slaughtering, adhering to the antemortem and postmortem inspection protocols with permission from the animal owners and under the Egyptian Guidelines for Bovine Inspection under The General Organization for Veterinary Services (GOVS): Law 517-year 1986.

### Parasite and sample collection

#### Tissue samples collection

During postmortem (PM) examination, fresh muscle samples (esophagus, tongue, masseter muscle and heart) were macroscopically inspected by specialized veterinary inspectors for the existence of cysts of *Sarcocystis* sp. Fresh muscle samples (2–3 mm^3^ in thickness) were obtained by a biopsy needle and forceps. Each sarcocyst was squeezed firmly between two glass slides and inspected under a microscope at a magnification of 10 × [14]. From each animal, about 60 − 75 *Sarcocystis* infected muscle samples were collected from 246 adult and old females and males water buffaloes (226 esophagus, 41 tongues, 9 masseter muscles and 3 hearts) in sterile labeled plastic bags and transported in an icebox to Immunology and Parasitology Laboratory, Veterinary Research Institute, National Research Centre (NRC), Egypt, for further examinations.

#### Serum samples

##### Serum samples from selected abattoirs

Nine hundred buffalo blood samples were collected in sterile glass tubes during slaughter from different abattoirs for seroprevalence study. Blood samples were transferred to our Lab., allowed to clot and then sera were separated and stored at − 20 °C until use.

##### Reference serum samples

Thirty-three serum samples from infection-free live buffalo calves (less than 1 year of age) which were considered as negative control samples and 48 serum samples from buffaloes naturally infected with other parasitic diseases: toxoplasmosis (*N* = 6) and cryptosporidiosis (*N* = 10), confirmed by PCR [1, 67], fasciolosis (*N* = 12), hydatidosis (*N* = 9), and paramphistomosis (*N* = 11), confirmed after PM inspection, but free from sarcocystosis were provided from samples of the research project (13,010,124), Veterinary Research Institute, NRC, under ethical approval protocol number 13010124.

#### Isolation of sarcocysts 

Individual sarcocysts were isolated from infected muscle tissues containing macrocysts. Morphological characteristics were examined by the naked eye and the *Sarcocysts* were extracted using forceps. Sarcocysts were then washed and cleaned with saline, kept at − 20 °C for molecular analysis and antigen preparation [13].

### Molecular identification

#### Genomic DNA extraction

Genomic DNA extraction from 70 cysts was performed using QIAamp DNA Mini kit (Qiagen, Germany) according to the manufacturer’s recommendations.

#### PCR amplification

The molecular identification of *Sarcocystis* spp. was performed through PCR targeting the* 18S* ribosomal RNA (rRNA) gene using primers (Metabion, Germany) as listed in Table 1. The reaction was performed according to Bahari et al. [11] in a BIO-RAD (Singapore) Thermal Cycler and the cycling conditions are provided in Table 1.

**Table 1:** Primer sequences, target gene, amplicon size and cycling conditions

Target gene	Primer sequences	Amplified segment (bp)	Primary Denaturation	Amplification (35 cycles)			Final extension
				**Secondary denaturation**	**Annealing**	**Extension**	
***Sarcocystis 18S* rRNA**	Sar-F1: 5'GCACTTGATGAATTCTGGCA3'	600	94˚C 5 min	94˚C 30 s	50˚C 40 s	72˚C 45 s	72˚C 10 min
	Sar-R1: 5'CACCACCCATAGAATCAAG3'						

#### DNA sequencing and phylogenetic analysis

The PCR products from 10 samples showing the sharpest bands were purified using gel purification kit (GeneDirex, NA006-0100, Taiwan) according to manufacturer instructions. Then, the purified PCR products were sequenced in an ABI 3130 DNA sequencer (Applied Biosystems, Japan) with the same primers which were used during PCR amplifications. The results were analyzed and corrected using ChromasPro 1.7 (Technelysium Pty Ltd., Australia). The obtained sequences were compared with those previously published in the NCBI using the nucleotide BLAST (http://www.blast.ncbi.nlm.nih.gov/Blast.cgi). The sequences were aligned using CLUSTALW version 1.83 in the MegAlign module (DNASTAR, Madison, WI, USA) and the phylogenetic analysis were performed using MEGA7 software [45].

### Histological examination of the sarcocyst

The 10% formalin, paraffin-embedded and dehydrated infected esophagus and tongue cyst samples were prepared and stained with hematoxylin and eosin (H&E) for cyst histological examination according to [34]. Stained slides were examined under CX41 Olympus (Japan) light microscope at 40 × magnification.

### Whole cyst antigen

#### Preparation of whole cyst antigen

The whole cyst antigen of *Sarcocystis fusiformis* recovered from tissues was prepared according to Gasbarre et al. [35] with some modifications. In brief, 50 cysts of *S. fusiformis* macrocysts (GenBank: PP336901.1 and PP336902.1) were collected into sterile tubes and washed thrice with 0.15 M sterile Phosphate Buffered Saline (PBS, pH = 7.2). The whole cysts were macerated in 5mL of PBS at room temperature until a cream-colored suspension was formed, and then homogenized by tissue homogenizer (Tenbroeck Tissue Grinder, PYREX®, USA) in PBS. The homogenate was freeze-thawed four times and sonicated using VCX750 Sonics Vibra Cell sonicator (Sonics & Materials Inc., USA) for 12 cycles/20 s each at 100 W. The suspension was centrifuged (Sigma 3-18KS 10370, Germany) at 12,000 × *g* for 30 min at 4 °C. The supernatant was collected and used as antigen and its protein concentration was estimated as per the method of Lowry et al. [49]. 2 mM Protease Inhibitor Phenyl Methyl Sulphonyl Fluoride (PMSF) at a concentration of 1 μl/mL of antigen and 0.02% NaN_3_ was added to prevent protein degradation. The antigen was aliquoted and stored at ‒20 °C till further use.

#### Hyperimmune rat sera

Ten Wistar rats (weighing 130‒150 gm) were used to raise the hyperimmune serum against the prepared antigen following the procedure described by Singh et al. [62] with some modifications. Briefly, rats were inoculated intramuscularly with 110 μg of whole cyst antigen of *S. fusiformis* after being emulsified with equal volume of Freund’s complete adjuvant (Sigma-Aldrich, USA). After primary immunization, the first booster dose was given intramuscularly on the14^th^ day, the second and third booster doses were given on 21 st and 28th day of the primary injection using Freund’s incomplete adjuvant (Sigma-Aldrich, USA). Blood samples were collected seven days after the last injection by puncturing the choroid plexus using fine-edge capillary tube. Sera were separated and the presence of specific antibodies was checked by indirect ELISA according to Savini et al. [59]. Serum samples were aliquoted and stored at ‒20 °C until use.

### Characterization of whole cyst antigen of *Sarcocystis fusiformis*

#### Sodium Dodecyl Sulfate–Polyacrylamide Gel Electrophoresis (SDS-PAGE)

The reducing 10% SDS-PAGE procedure was utilized to assess the molecular mass of the prepared whole cyst antigen of *S. fusiformis* [46]. The antigen and Prestained Protein Ladder (GeneDirex BLUltra, USA) were separately electrophoresed on the same gel of SDS-PAGE. The slab gel after separation was stained with Coomassie Brilliant Blue dye, photographed, and analyzed using Image Lab Software (Gel Doc™ XR^+^, Bio-Rad, USA).

#### Western blotting

Western blot analysis was performed to determine the immunogenic protein (polypeptides) of whole cyst antigen that reacts against specific antibodies (IgGs) of *S. fusiformis* infection as described by Towbin et al. [66]. Briefly, after electrophoresis, gel was blotted onto nitrocellulose membranes (Himedia, USA) in a blotting system. After blocking and washing, membranes were incubated with pooled positive naturally infected buffalo serum, hyperimmune rat serum and negative control sera (buffalo and rat), separately. Prestained protein marker (GeneDirex BLUltra, USA) was also loaded. Anti-bovine IgG horseradish peroxidase conjugate (Sigma-Aldrich), Anti-Rat IgG horseradish peroxidase conjugate (Sigma-Aldrich) and 4-chloro-1-naphthol (Santa Cruz Biotechnology, USA) were used. Finally, the membrane was photographed and analyzed using Gel Doc™ XR^+^ with Image Lab Software (Bio-Rad, USA).

### Serodiagnosis of sarcocystosis

#### Indirect ELISA

Indirect ELISA was adopted to define the activity of the whole cyst antigen of *S. fusiformis* in detecting anti-*Sarcocystis* IgG in 246 serum samples from *Sarcocystis-*positive buffaloes as examined at PM (considered as control positive) and reference sera [33 healthy negative buffalo sera (control negative), and 48 serum samples of buffaloes naturally infected with other parasites but not *Sarcocystis*] and calculate its immunodiagnostic values (specificity, sensitivity, positive and negative predictive values (PPV and NPV), and diagnostic accuracy percentages) using equations according to Zane [68] and Thrusfield [65]. In addition, indirect-ELISA was used to detect anti-*S. fusiformis* antibodies for diagnosis of sarcocystosis and calculation of the infection rate percentage in the collected buffaloes'serum samples (*N* = 900).

#### Standardization of indirect-ELISA

The working dilution and concentration of whole cyst antigen (4 µg/well), dilution of sera (1:100), Anti-bovine IgG horseradish peroxidase conjugate (Sigma-Aldrich; 1:1000), and dilution of Anti-Rat IgG horseradish peroxidase conjugate (Sigma-Aldrich; 1:1000) were determined by checkerboard titrations [61, 67]. The optical densities (OD) were read at 450 nm using ELISA reader (BIO-TEK, INC., ELx, 808 UV). The cut off value was calculated by mean OD values of negative control + 2 Standard Deviation, SD [2].

### Statistical analysis

Statistical analyses were conducted using SPSS version 20 for Windows (IBM Corp., Armonk, NY, USA). Data were tested for normality via the Kolmogorov–Smirnov test of normality, which outcomes indicated that data were normally distributed. The diagnostic accuracy parameters of the test were evaluated by calculating the sensitivity, specificity, NPV, PPV, diagnostic accuracy, area under the curve (AUC), receiver operating characteristic (ROC) curve, and Chi square using SPSS software. The AUC values were evaluated according to Swets [63] as follows: AUC < 0.5 (Noninformative), 0.5 < AUC < 0.7 (low accuracy), 0.7 < AUC < 0.9 (moderate accuracy) and 0.9 < AUC < 1 (high accuracy). *P* < *0.05* was considered statistically significant.

## Results

### PM infection survey of* Sarcocystis cysts* in water buffaloes

**Fig. 2:**
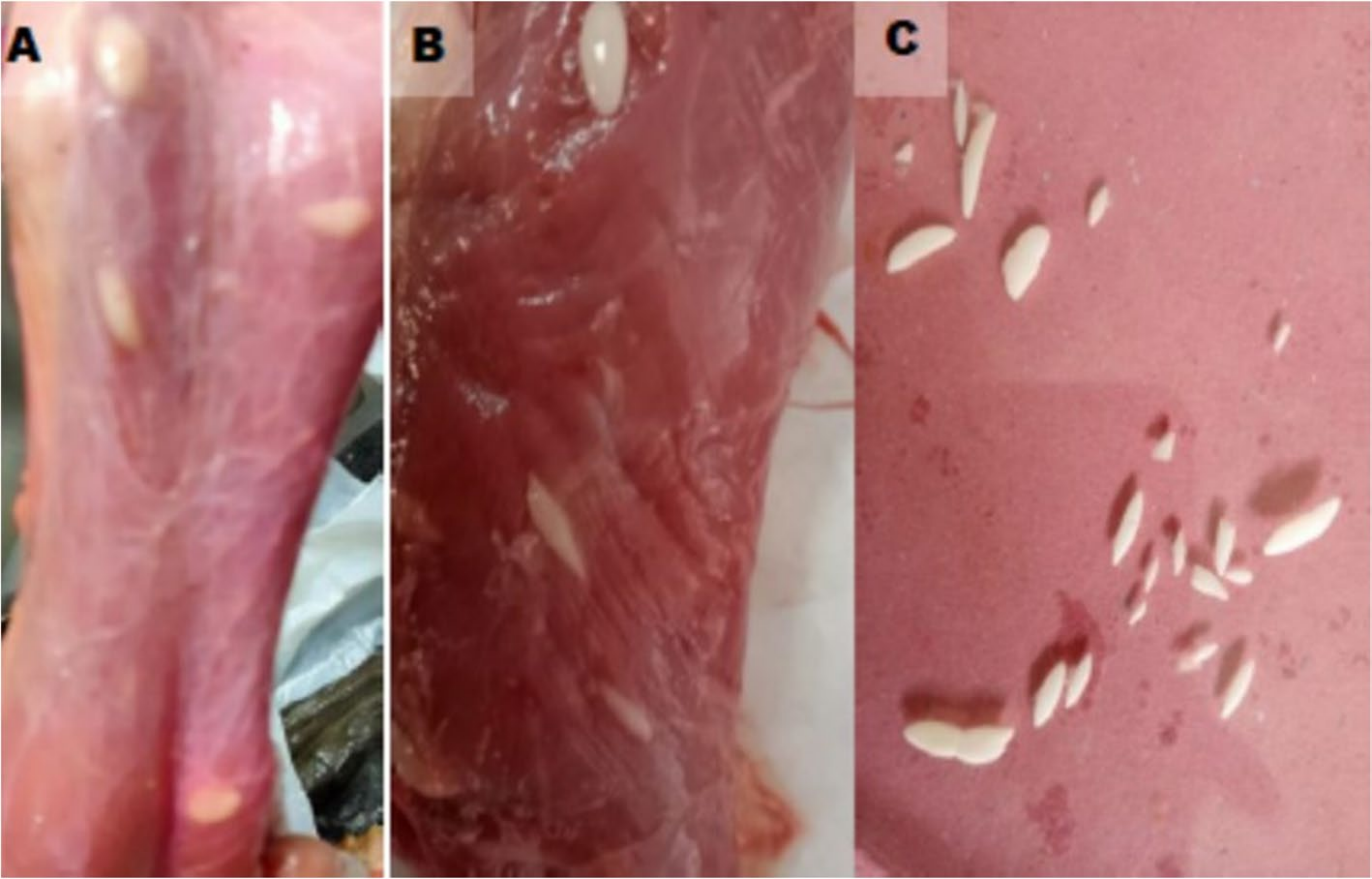
Macroscopical *Sarcocystis fusiformis* from water buffaloes, spindle-shaped milky white *Sarcocystis* embedded in esophagus (**A**), masseter muscle (**B**) and stripped *Sarcocystis* (**C**)

Morphological characteristics of macrocysts based on macroscopic examination showed the cysts with whitish, fusiform or spindle-shaped structures embedded within the muscle tissues of the esophagus, masseter muscles, tongue and heart. They were also found opaque, milky white in color, and varied in size, measuring approximately 4–16 mm in length and 1–5 mm in width (Fig. 2: A, B and C). Small muscle tissue pieces were compressed between two slides and examined under microscope and no microscopic cysts were detected.

The total sarcocystosis infection among examined slaughtered buffaloes was 246 out of 900 (27.3%). The infection rate was highest in El-Beheira (47.4%) followed by Cairo (30.7%) then Giza (19.5%) Governorates (Table 2).

**Table 2:** Infection surveillance of macroscopic *Sarcocystis* spp. in buffalo carcasses from Egyptian abattoirs

Variables		No. of examined buffaloes	No. of *Sarcocystis* positive	Infection rate (%)
**Locality**	Cairo	300	92	30.7%
	Giza	200	39	19.5%
	Beni-Suef	60	6	10.0%
	Al-Sharqia	70	12	17.1%
	Qalyubia	80	7	8.8%
	El-Beheira	190	90	47.4%
**Sex**	Male	400	55	13.8%
	Female	500	191	38.2%
**Age**	Young (< 2 years)	100	0	0%
	Adult (2‒5 years)	340	60	17.6%
	Old (5‒10 years)	460	186	40.4%
**Organs**	Esophagus	900	197/246	80.1%
	Tongue		15/246	6.1%
	Masseter muscle		2/246	0.8%
	Heart		3/246	1.2%
	Esophagus and Tongue		22/246	8.9%
	Esophagus and Masseter muscle		3/246	1.2%
	Esophagus, Tongue and Masseter muscle		4/246	1.6%

According to age, the infection rate was higher (40.4%) in old buffaloes than in adult buffaloes (17.6%), and no macroscopic sarcocysts were detected in young buffaloes. Additionally, PM survey of *Sarcocystis* infection in old females was higher than in males (38.2% and 13.8%, respectively) (Table 2).

The results of the PM examination showed that the esophagus muscle was recorded as the most susceptible organ to macrocysts of *Sarcocystis* (80.1% in esophagus muscle only and 8.9%, 1.2% and 1.6% for prevalence in esophagus and tongue, esophagus and masseter muscle, and esophagus, tongue and masseter muscle, respectively) while the prevalence in tongue muscle only was 6.1%. A very low infection was observed in the masseter muscle and heart (0.8% and 1.2%, respectively; Table 2).

### Molecular identification of *Sarcocystis* spp.

**Fig. 3:**
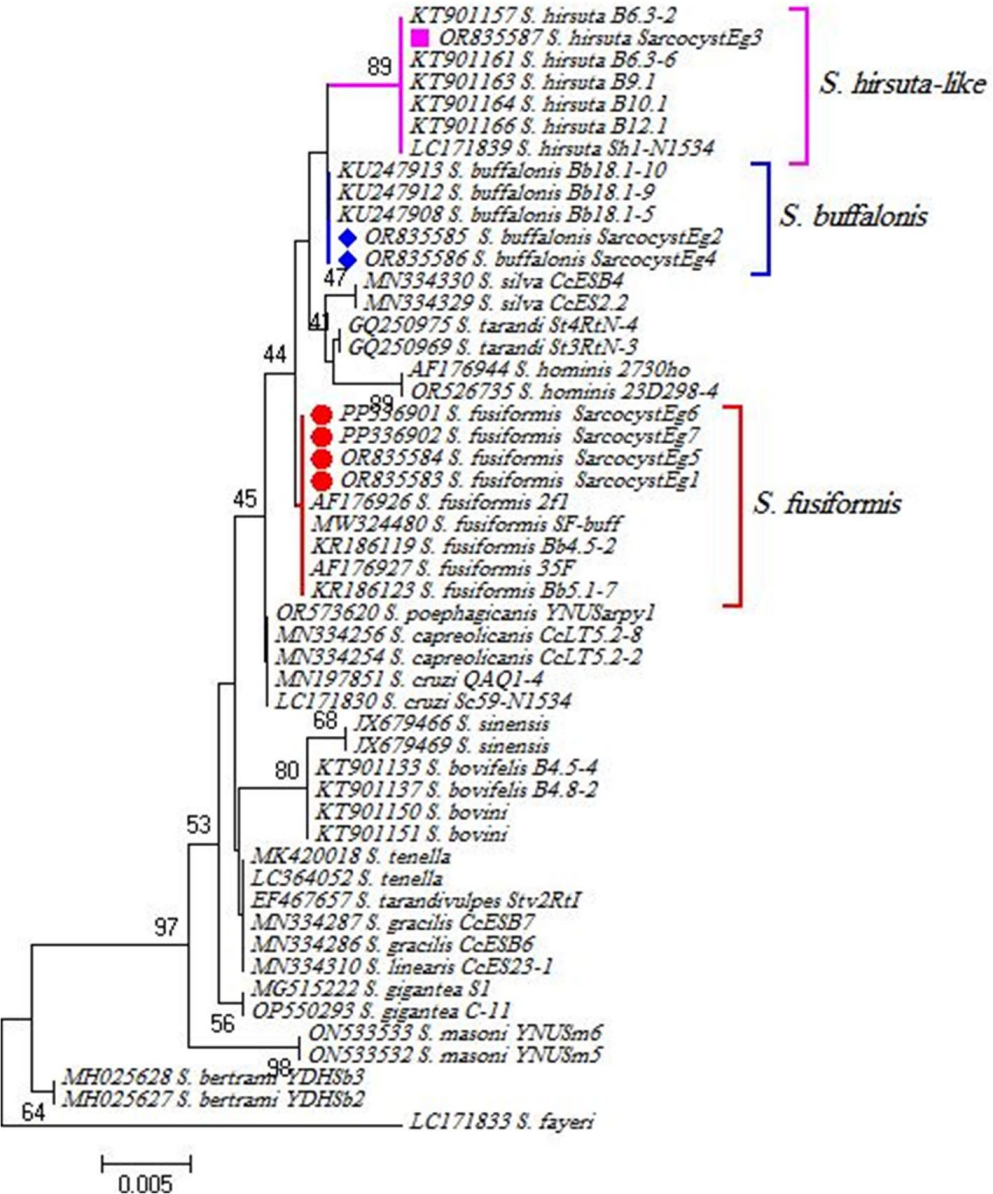
Phylogenetic analysis using the maximum likelihood method based on *18S* rRNA gene for* Sarcocystis* sp. Isolate sequences obtained in this study are highlighted: pink square for *S. hirsuta-*like (GenBank: OR835587.1), blue diamonds for *S. buffalonis* (GenBank: OR835585.1 and OR835586.1) and red dots for *S. fusiformis* (GenBank: OR835583.1, OR835584.1, PP336901.1 and PP336902.1), Our genotype clustzered with other *Sarcocystis* sp. references. The scale bar represents a 5% nucleotide sequence divergence

The *18S* rRNA gene sequencing confirmed the presence of three *Sarcocystis* species from buffaloes (7 isolates). The first species was *S. hirsuta-*like isolated from esophagus from Cairo (GenBank: OR835587.1) which showed 100% identity (483/483) with *S. hirsuta* detected in skeletal muscles of cattle (*Bos taurus*) from New Zealand (GenBank: KT901157.1, KT901161.1, LC171839.1). The second species was *S. buffalonis* which was isolated from tongue from Al-Sharqia (GenBank: OR835585.1) and esophagus from Beni-Suef (GenBank: OR835586.1) which showed 100% identity (483/483) with *S. buffalonis* detected in esophagus of buffalo (*Bubalus bubalis*) from Egypt (GenBank: KU247913.1, KU247903.1, KU247901.1). We also isolated four *S. fusiformis* isolates from esophagus from Giza (GenBank: OR835583.1), *S. fusiformis* from tongue from Qalyubia (GenBank: OR835584.1) and *S. fusiformis* from esophagus from El-Beheira (GenBank: PP336901.1 and PP336902.1) which showed 100% identity (480/480) water buffalo (*B. bubalis*) esophagus (GenBank: KR186119.1, KR186123.1) and muscles (GenBank: MW324480.1) from Egypt and buffalo muscles from China (GenBank: AF176926.1 and AF176927.1). Phylogenetic analysis of indicated that our sequences clustered within the same clade as reference species (Fig. 3).

### Histological findings

**Fig. 4:**
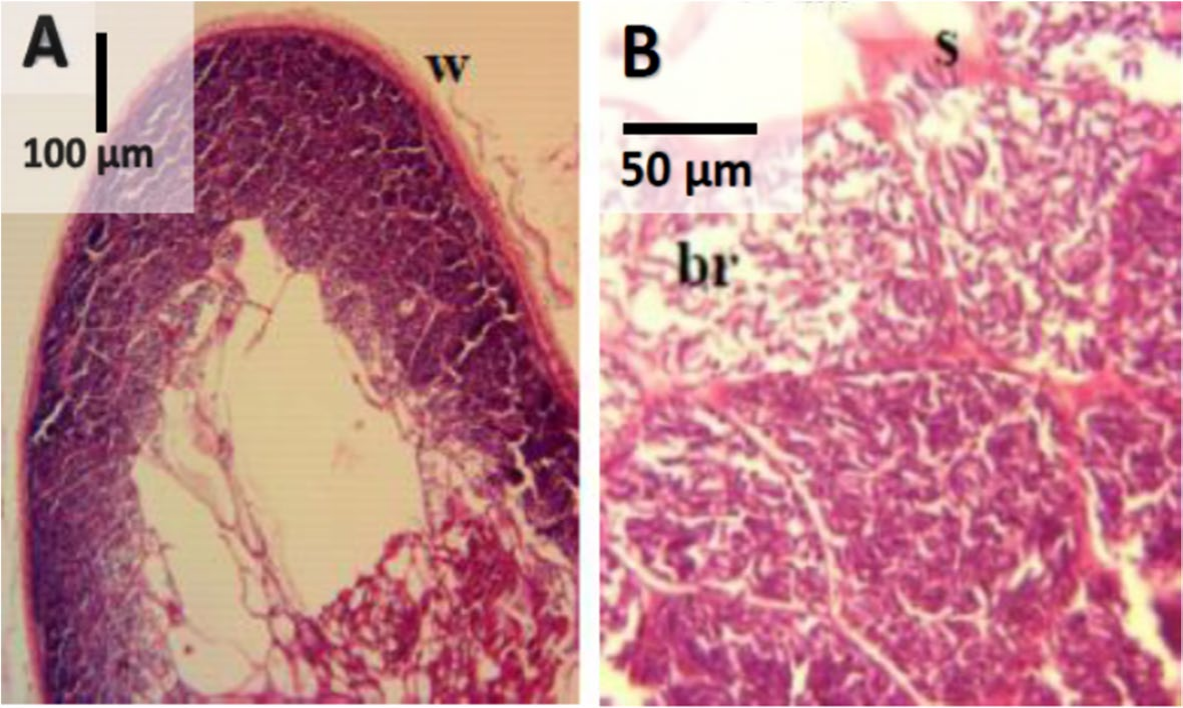
Histological section of *Sarcocystis* macrocyst stained with H&E, × 40 (**A**) and × 400 (**B**). *Sarcocystis* showed thin wall (w), septa (s) and clusters of bradyzoites (br)

The H&E staining sections revealed irregular shaped *Sarcocystis* observed within the muscle fibers of the esophagus and tongue muscles. A smooth, thin cyst wall surrounded the sarcocyst, and that thin septum separated the cyst cavity into several compartments of varying sizes. Within the sarcocysts were two parasite stages: metrocytes, which were found close to the cyst's edge and stained palely, and the cylinder, curving basophilic bradyzoites, which occupied most of the cyst and were densely packed in its interior, displaying a dark stain (Fig. 4: A & B).

### Characterization of whole cyst antigen by SDS-PAGE gel electrophoresis

**Fig. 5:**
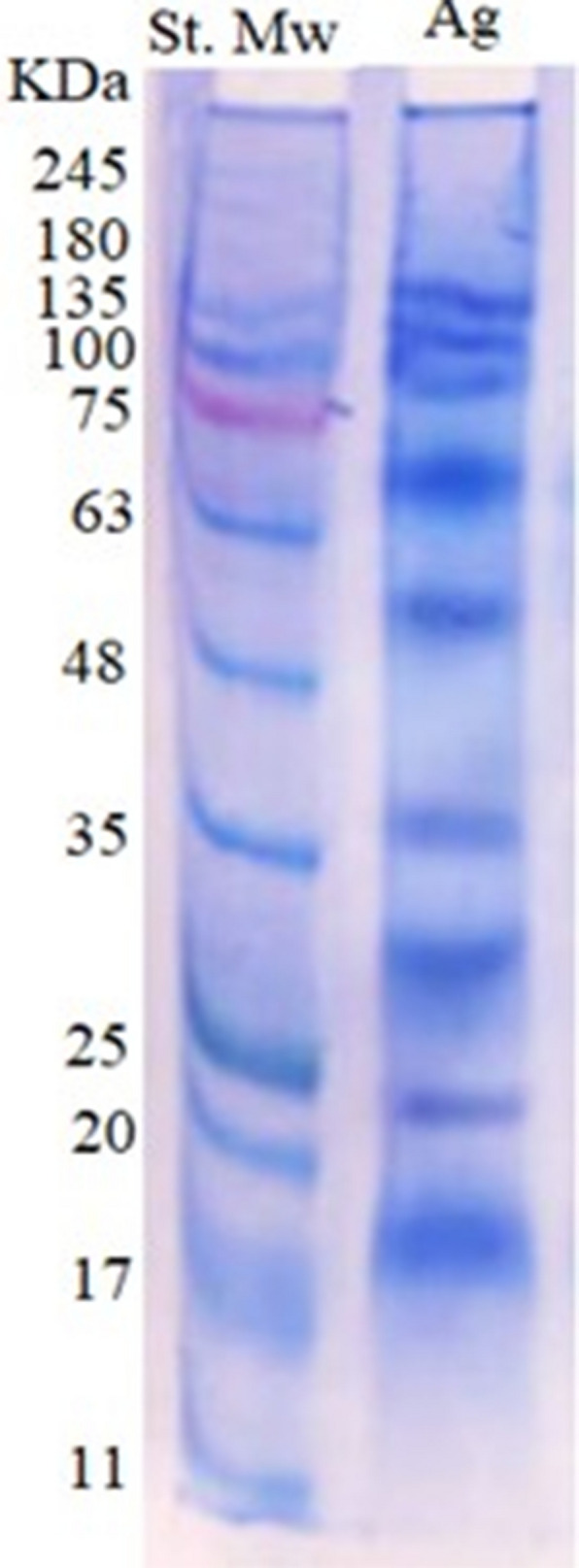
Electrophoresis profile of whole cyst antigen of *S. fusiformis* (Lane Ag) and Prestained protein molecular weight marker (Lane St. Mw.)

The protein concentration of the whole cyst antigen prepared of *S. fusiformis* was 973 µg/ml. The electrophoresis profile of whole cyst antigen showed nine major polypeptides ranging from 140 to 19 kDa. The major bands were detected at molecular weights 140, 120, 78, 66, 53, 39, 32, 24, and 19 kDa (Fig. 5).

### Immunoreactive peptides detected in whole cyst antigen

**Fig. 6:**
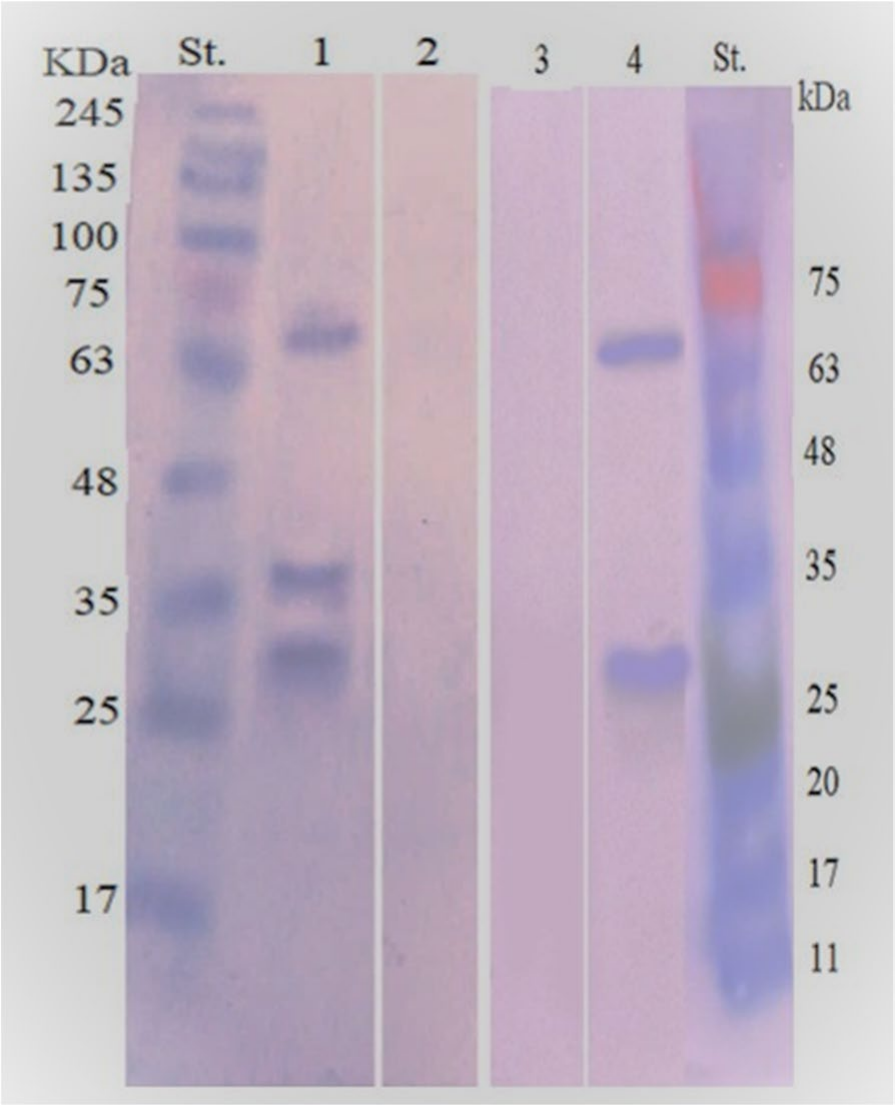
Western blot immunoreactive bands of whole cyst antigen of *S. fusiformis* identified by sera from *S. fusiformis* naturally infected buffalo sera (Lane 1), hyperimmune rat sera (Lane 4), negative control buffalo sera (Lane 2), and negative control rat sera (Lane 3). Prestained molecular weight marker was loaded in Lane St

Western blot analysis identified three immunogenic reactive bands of molecular weights 66, 39, and 32 kDa with pooled *S. fusiformis* naturally infected buffalo serum samples (Lane 1), and only two immunogenic reactive bands of molecular weights 66 and 32 kDa were observed with pooled hyperimmune rat sera with whole cyst antigen of *S. fusiformis* (Lane 4)*.* No immunoreactive polypeptides were identified in negative control buffalo sera (Lane 2) and negative control rat sera (Lane 3). This analysis for immunoreactivity peptides confirmed a 100% specificity of prepared whole cyst antigen (Fig. 6).

### Reactivity, sensitivity and specificity of whole cyst antigen for detection of anti-*Sarcocystis* antibodies in buffalo sera by Indirect-ELISA

The mean OD values of IgG in *S. fusiformis* naturally infected buffalo sera were 1.251 ± 0.360 (mean OD ± SD**)** which were significantly higher (*P* > *0.01*) than both the negative control group (0.255 ± 0.069) and the other parasitic diseases groups (0.260 ± 0.0198). No cross-reactions were recorded with sera of buffalo infected with other parasitic diseases or with negative control sera. Consequently, all the 33 negative control serum samples and 48 samples of buffalo sera infected with other parasitic diseases group were recorded OD values below OD of cut-off value (0.3865), recording a specificity of 100%. The Area under ROC curve (AUC) was 0.994 (*P* < *0.001*) recording high accuracy potential (0.9 > AUC > 1), Confidence Interval was 0.998 and 1.001, and Chi-Square = 279 (*P* < *0.001*). About 240 samples out of 246 positive control serum samples were detected as *Sarcocystis* positive samples recorded a sensitivity of the assay 97.6%, with a diagnostic accuracy of 98.5% as immunodiagnostic values shown in Table 3.

**Table 3:** Immunodiagnostic values in the detection of anti-*Sarcocystis* antibodies in buffalo serum by Indirect-ELISA

Sensitivity	Specificity	PPV	NPV	AUC/*P*	X^2^/*P*	Diagnostic Accuracy
97.6%	**100%**	**100%**	**93.1%**	0.994/*P* < 0.001	279/*P* < 0.001	**98.5%**

*PPV* Positive Predictive Value, *AUC* Area Under Curve, *X*^*2*^  Chi Square, *NPV* Negative Predictive Value

*P* < 0.001: Highly Significant

### Seroprevalence of *Sarcocystis* infection in buffalo sera by indirect ELISA

**Fig. 7:**
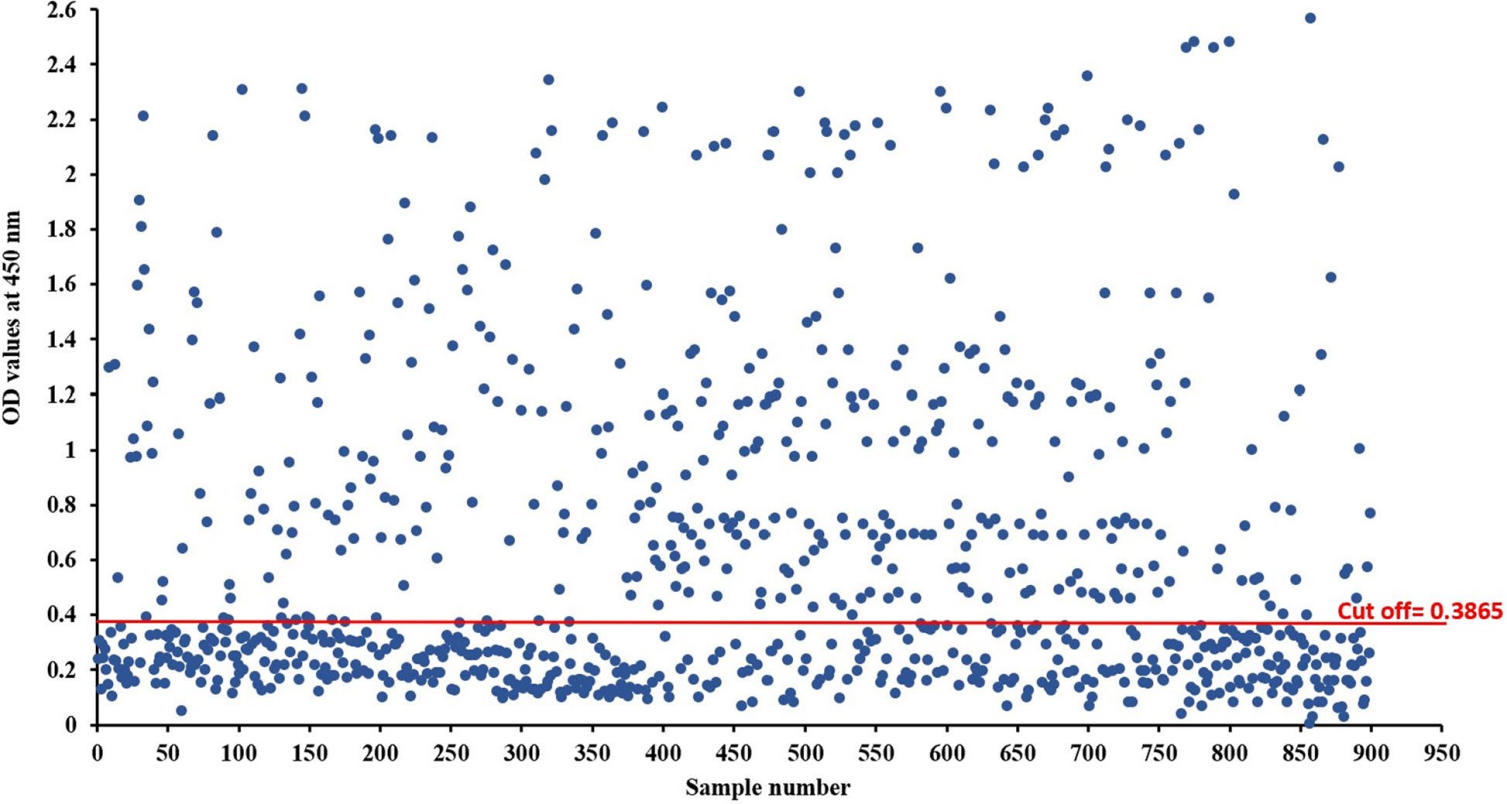
Detection of specific IgG antibodies against *S. fusiformis* by indirect ELISA. The figure illustrates the elevation in the level of IgG within naturally infected buffaloes compared to non-infected ones. The cut-off = 0.3865

The detection of specific IgG antibodies against *S. fusiformis* infection by indirect ELISA showed that among 900 tested samples about 418 (46.4%) revealed positive ODs for IgG antibodies (Fig. 7). The cut-off for positivity was 0.3865.

## Discussion

Sarcocystosis is a common zoonotic protozoan disease caused by *Sarcocystis* sp. infecting humans and domestic animals such as cattle, and pigs [19, 26]. In the current study, the overall infection rate of suspected sarcocystosis by visual macroscopic inspection was 27.3% with the highest infection rates recorded in El-Beheira and Cairo abattoirs. According to El-Sayad et al. [28], the highest infection rates might be due to the climatic conditions of the Northern Delta of Egypt which are favorable for the survival of *Sarcocystis* spp. In this region, warm, humid environments, abundant water sources and agriculture prosperity might create a perfect environment for the parasite and its intermediate hosts (farm animals) and definitive hosts (dogs and cats which present close to livestock in the Delta region) and facilitate *Sarcocystis* transmission cycle. Similar results of macroscopic prevalence of sarcocystosis among slaughtered buffaloes were recorded in Egypt [3, 10, 36, 50, 55], 52% in Sohag [44] and 71.5% in Assiut [60]. On the other hand, higher infection rates were recorded among other countries reaching 65% in The Philippines [47] and 100% in Argentinean and Nigerian cattle [51, 56]. These differences might be due to the assumption that the locality significantly affects the spread of infection because of different husbandry management systems [38], close contact between the final and intermediate hosts and environmental contamination [18, 25], geographical distribution [10].

In our study, depending on age, three categorized groups of naturally infected buffaloes were examined. Among these groups, sarcocysts were most common in adult buffaloes and the infection rate increased by age (5 − 10 years). Our results agreed with El-Sayad et al. [28] who recorded a 21.9% infection rate in the age group 2 − 5 years, and 48.5% among the age group 5 − 10 years, Ibrahim et al. [41], Ras et al. [58] and Elbarbary et al. [24] who demonstrated a higher prevalence of sarcocystosis in older animals. Also, Elsharkawy et al. [30] found that the prevalence of *Sarcocystis* sp. is based on the age of carcasses where adults had a higher infection rate (78.5%) compared to (59.8%) in young ones. Similarly, a gradual increase in infection with age was reported in previous studies in Egypt [23, 34, 36, 50] and other countries, [29, 31, 53]. These results are most likely due to longer and repeated exposure to the infection in aged buffaloes and repeated exposure which gradually accumulates cysts in muscles [5, 64]. In addition, cysts need longer time to appear macroscopically compared to microscopic cysts [47, 52].

In our study, most of infected females were more than five years old, this finding agreed with El-Sayad et al. [28] who recorded that all infected females were > 5 years old. Close to these results, Ibrahim et al. [41], Gerab et al. [36], Elsharkawy et al. [30], Ali et al. [7] and Elbarbary et al. [24] reported a higher prevalence of sarcocystosis in female buffaloes (37, 42, 84.7, 62.8 and 51.6%, respectively) than males (11, 27, 57.8, 10.5 and 20.3%, respectively). A possible explanation for this higher infection rates in females could be due to stress factors weakening their immune system such as pregnancy, gestation and lactation [41]. In addition, the low percentage of infected males might be attributed to the animal management system as most of the males are kept only for the fattening system and slaughtering at approximately 2 years of age, on the contrary, females are kept for long times for milk production [27].

Since muscles from the esophagus, heart, tongue and diaphragm are the target-colonizing areas of* Sarcocystis* sp. [19], we found the highest numbers of macrocysts in the esophagus whereas the least macrocysts were recorded in the heart after PM examination. Same results were recorded in buffaloes in Egypt by Gerab et al. [36] who demonstrated that esophagus was the most affected organs with *Sarcocystis* (53.5%) followed by the tongue (45.5%). Also, previous studies recorded the esophagus to be as the most affected organ, [7, 24, 28, 29, 43]. This might be explained by the presence of esophagus as an earlier stop in the migration route of *Sarcocystis* sp. [24]. It may be related to the type of esophageal muscle tissue present. Unlike other organs, such as the heart and skeletal muscles, which are composed of denser and more compact muscle fibers, the esophageal muscle, primarily composed of smooth muscle, has looser and more delicate structure. This may facilitate the development and enlargement of macroscopic cysts.

Molecular diagnostics have been used for specific identification of *Sarcocystis* spp. The *18S* rRNA gene was shown to be a good genetic marker for the species-specific recognition of *Sarcocystis* spp. [42]. For accurate species identification, we amplified a partial sequence of *18S* rRNA gene from DNA extracted from 70 tissue cysts samples to confirm species recognition. We isolated three *Sarcocystis* species from buffaloes: *S. hirsuta-*like, *S. buffalonis* and mostly *S. fusiformis* highlighting the diversity of species circulating in Egyptian buffaloes. These results aligned with previous studies which recorded that the most common species of *Sarcocystis* in naturally infected water buffaloes in Egypt was *S. fusiformis* [40, 48] and that *S. fusiformis*, *S. buffalonis*, *S. levinei*, *S. dubeyi*, and *S. sinensis* are the main species infecting water buffaloes [48]. Notably, to the best of our knowledge, this study might represent the first detection of *S. hirsuta-*like in buffaloes in Egypt, which is common species infecting cattle [21]. To strengthen the findings, generating more sequences using the *18S* rRNA gene and additional genetic markers (such as: *COX1* and *ITS* genes) would be beneficial for validation.

In the present study, macroscopic cysts appeared spindle shape, creamy color and easily isolated from infected muscles mass and not damaged by freezing. Comparable description of *Sarcocystis* cysts was published elsewhere [20, 25, 50, 54]. In the current study, we found that although there was an infiltration of inflammatory cells surrounding solitary *Sarcocystis*, there were no signs of inflammatory or degenerative changes. This observation aligns with the theory that the protozoan *Sarcocystis* is found enclosed by a cyst inside muscle fibers which provides a defense from host immunity. These findings coincide with previous studies [24, 34] who didn’t find any signs of inflammation in the tissue surrounding the cysts.

Sarcocystosis is difficult to be detected in live animals and the serological tools could help in antemortem diagnosis. Thus, in this study we developed an indirect ELISA based on the whole cyst antigen of *S. fusiformis.* The protein profile of this antigen using SDS-PAGE was revealed that 9 major polypeptides (140, 120, 78, 66, 53, 39, 32, 24, and 19 kDa) were observed. Using western blotting, 3 immunogenic reactive bands (66, 39, and 32 kDa) were identified, where the major common immunoreactive polypeptides were 66 and 32 kDa. Our results were almost similar to polypeptides observed by Singh et al. [62] who carried out electrophoretic separation of whole cyst lysate antigen of *S. fusiformis* showing 12 polypeptides (78, 66, 53, 50, 42, 39, 32, 29, 27, 24, 19 and 15 kDa), and after conducting western blotting, six (32, 39, 42, 53, 66, 78 kDa) and two (53, 66 kDa) polypeptides were found to be immunoreactive with hyperimmune rat sera and positive natural buffalo sera. Differences in banding profiles could be due to type of antigen or its preparation method. Also, the percentage of resolving gel might affect the separation of polypeptides.

Indirect ELISA is considered a safe and eco-friendly, simple, highly specific and sensitive diagnostic assay [6]. Our results revealed that 46.4% were seropositive to sarcocystosis by indirect ELISA with a specificity of 100% and sensitivity of 97.6% for detection of antibodies against *Sarcocystis* spp. A previous study of Ashmawy et al. [8] reported a sensitivity of 94%, specificity of 100% and seropositivity of 67.6%. Moreover, Ferreira et al. [32] detected *Sarcocystis* sp. antibodies in 96% and 80% of cattle serum samples at 1:25 and 1:200 dilutions, respectively. Additionally, Metwally et al. [50] found that a positive rate for sarcocystosis was (94.5%) using ELISA. ELISA showed a great value in evaluating a large number of samples and detecting both acute and chronically infected live animals showing clinical signs or not [33].

## Conclusion

The obtained results of this study confirmed the high incidence of sarcocystosis in female buffaloes than males, and in old animals than young animals. We identified *S. buffalonis*, *S. fusiformis* and *S. hirsuta-*like in buffaloes from different Governorates of Egypt highlighting the diversity of species circulating in Egyptian buffaloes. Further molecular analysis based on *COX1* and *ITS* region are required for the exact characterization of species identified in the present study. Using ELISA in the detection of *Sarcocystis* antibodies in animals is recommended for routine examination to effectively control this disease in animals and avoid human infection.

## Supplementary Information

Supplementary Material 1.

Supplementary Material 2.

Supplementary Material 3.

Supplementary Material 4.

Supplementary Material 5.

## Data Availability

All data generated and analyzed in this study are included in the published manuscript. The nucleotide sequences of S. hirsuta-like, S. buffalonis and S. fusiformis for the 18S rRNA gene obtained in this study have been submitted to the GenBank, GenBank accession numbers: OR835587.1, OR835585.1, OR835586.1, OR835583.1, OR835584.1, PP336901.1 and PP336902.1 (https://www.ncbi.nlm.nih.gov/genbank).

## Abbreviations

*18S * rRNA: *18S *ribosomal RNA gene
AUC: Area Under Curve
CI: Confidence Interval
ELISA: Enzyme Linked Immunosorbent Assay
GOVS: General Organization for Veterinary Services
H&E: Hematoxylin and Eosin
IgG: Immunoglobulin G
kDa: Kilodalton
NPV: Negative Predictive Value
OD: Optical Density
PBS: Phosphate Buffered Saline
PCR: Polymerase Chain Reaction
PM: Postmortem
PPV: Positive Predictive Value
ROC: Receiver Operating Characteristic
*S.*: *Sarcocystis*
SD: Standard Deviation
SDS-PAGE: Sodium Dodecyl Sulfate Polyacrylamide Gel Electrophoresis
sp.: species
y.: Years old
