# Supplementary figures and images for: Sarcocystis species: molecular identification and seroprevalence in water buffaloes (Bubalus bubalis)
Source: BMC Vet Res. 2025 Jul 22;21:482. doi: 10.1186/s12917-025-04933-3 (PMC12281994; doi:10.1186/s12917-025-04933-3)

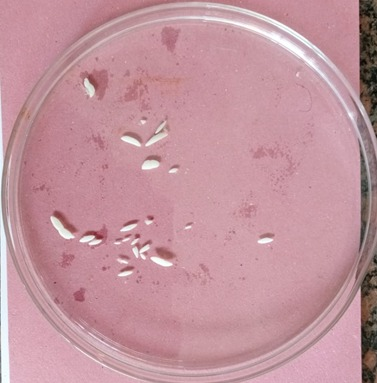

Supplement: Supplementary file 1 — Supplementary Material 1. [file 12917_2025_4933_MOESM1_ESM.tif]

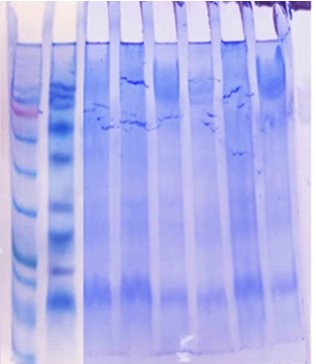

Supplement: Supplementary file 2 — Supplementary Material 2. [file 12917_2025_4933_MOESM2_ESM.jpg]

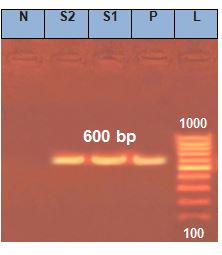

Supplement: Supplementary file 3 — Supplementary Material 3. [file 12917_2025_4933_MOESM3_ESM.jpg]

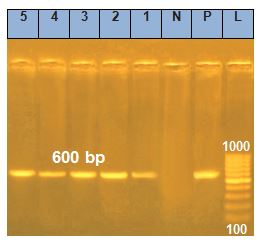

Supplement: Supplementary file 4 — Supplementary Material 4. [file 12917_2025_4933_MOESM4_ESM.jpg]

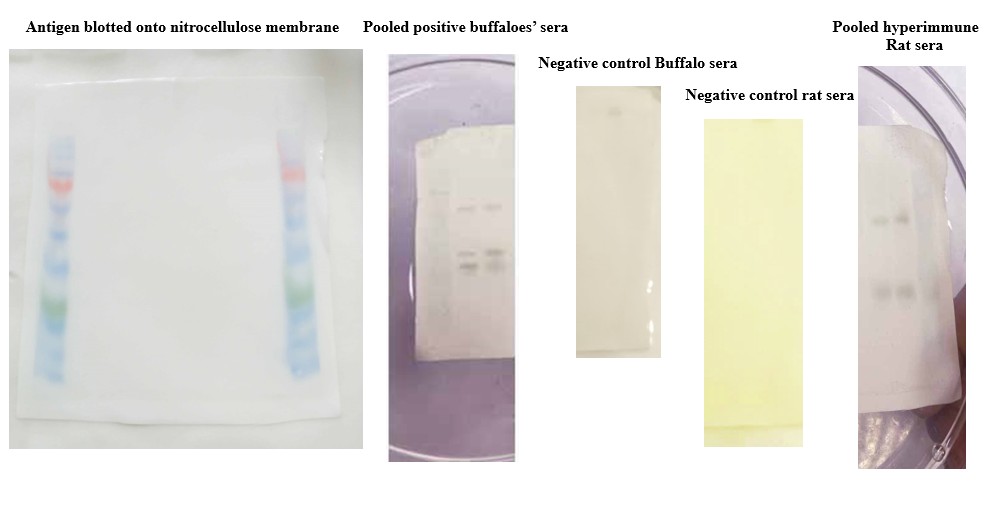

Supplement: Supplementary file 5 — Supplementary Material 5. [file 12917_2025_4933_MOESM5_ESM.jpg]
